# Quantification of the Synergism Between HER-Targeted Drugs with Human Blood Serum and EGF

**DOI:** 10.32607/actanaturae.27649

**Published:** 2026

**Authors:** D. E. Kamashev, E. M. Leboshchina, A. V. Koleboshina, I. A. Lavrinenko, G. A. Vashanov, Yu. D. Nechipurenko

**Affiliations:** Shemyakin–Ovchinnikov Institute of Bioorganic Chemistry, Moscow, 117997 Russia; World-Class Research Center “Digital Biodesign and Personalized Healthcare”, Sechenov First Moscow State Medical University, Moscow, 119991 Russia; Moscow Institute of Physics and Technology, Dolgoprudny, 141701 Russia; Voronezh State University, Voronezh, 394018 Russia; Engelhardt Institute of Molecular Biology, Moscow, 119991 Russia

**Keywords:** targeted therapy, HER2, breast cancer, human blood serum, trastuzumab, lapatinib

## Abstract

Finding the optimal combination of drugs for the effective inhibition of cancer cell growth is an extremely important task today, as the number of such drugs continues to grow. There are several approaches to determining the nature of drug interactions, allowing one to establish whether they are additive, synergis tic, or antagonistic. One such approach is described here, and it is demonstrated how to quantitatively meas ure the degree of interaction between two drugs. It is shown that human peripheral blood serum and EGF modulate the activity of HER2-targeted drugs in inhibiting the proliferation of HER2-positive BT474 and SK-BR-3 cells. We compared the effect of blood serum samples from breast cancer (BC) patients and healthy donors on the action of trastuzumab. Using the proposed method, it is possible to calculate the Combination Index (CI). For 17 serum samples from healthy donors, the mean CI was 0.396, while for 19 serum samples from patients with BC, the mean CI was 0.214. These results indicate a synergistic interaction between tras tuzumab and blood serum in both groups. We also found significant differences in CI values between healthy donors and breast cancer patients: blood serum samples from patients enhance the effect of trastuzumab to a greater extent.

## INTRODUCTION

**Theoretical foundations and mathematical approaches to describing experimental results**

1. Hill equation. Back in the 19th century, the law of mass action was proposed. It found wide applica tion in enzymology, pharmacology, toxicology, epide miology, sociophysics, and other fields [1, 2]. Based on this law, in his study of the oxygen transport function of blood, G. Hüfner (1890) proposed the first equa tion for hemoglobin oxygenation [3]. In enzymology, L. Michaelis and M. Menten (1913) formulated the basic equation of enzyme kinetics [4] and in physics, I. Langmuir (1916) presented a similar equation for monomolecular adsorption [5]. These equations can be reduced to the following general form:

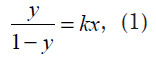



or alternatively:

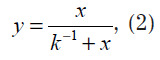



where k is the equilibrium constant of the reaction.

It should be noted that k^−1^ is a constant that has different letter designations in the corresponding equations in different fields of research; k^−1^ is the value of the argument x at which y takes the value ½ (y = 1 − y, y = 0.5, when x = k^−1^).

Analysis of the oxygen hemoglobin dissociation curves showed that G. Hüfner’s equation cannot satis factorily describe the experimental data. A. Hill (1910) then proposed an empirical equation of the following form [6]:

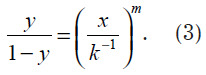



This equation can also be rewritten as:

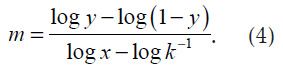



As follows from Eqs. (1) and (2), the Hill equation is related to the law of mass action, where the ex ponent m formally indicates the order of the reac tion. However, in the description of the experimental data, this parameter takes non-integer values [6, 7, 8]. Subsequently, this discrepancy was explained by in troducing the concept of a cooperation coefficient, which links the number of interacting subunits and the degree of their coordination during the interaction of ligands with a protein macromolecule [9].

An important advantage of the Hill equation is the simplicity of calculating its parameters from experi mental data. This has allowed researchers to apply the equation to a wide range of problems: from de scribing the process of oxygenation and enzyme ki netics (by refining the Michaelis–Menten equation) to studying dose–effect relationships in pharmacology [10, 11, 12, 13].

2. The Chou median-effect equation. The Hill equation is successfully used in pharmacokinetics and phar macodynamics [14, 15]. The dose–response curve is described accurately by this equation. In this case, x is the concentration or dose of the active substance D (drug, substrate, agonist, inhibitor, toxin, poison, me dicinal substance, etc.); and k^−1^ is the half-maximal (median) effective concentration (EC_50_ ), half-maximal inhibitory concentration (IC_50_ ), or effective (half-effec tive) dose (ED50 ) [16, 17].

Later, Chou and Talalay systematized the relation ships based on the law of mass action (Henderson Hasselbach, Hill, Michaelis–Menten, Scatchard), ob taining a unified form of recording relationships known as the median-effect equation [18, 19, 20]. In this equation, x = D, k^−1^ is denoted as Dm (median-effect dose), or the dose (concentration) that causes the me dian effect; y = f_a_ (fraction affected) is the proportion (of targets) exposed to the drug; 1 – y = f_u_ (fraction unaffected) is the proportion not affected by the drug, and m is a parameter characterizing the shape of the dose–response curve. The Hill equations (3) and (4) can be reduced to the median-effect equation by ex pressing it as the ratio of fa to fu (Eq. (5)) and calculat ing D (Eq. (6)) and fa (Eq. (7)):

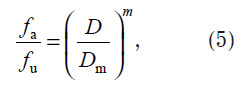

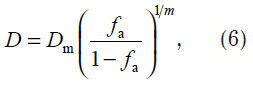

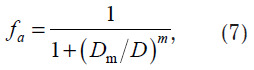







and then logarithmize to equality:

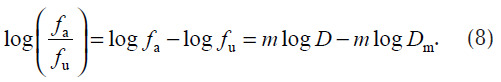



Therefore:

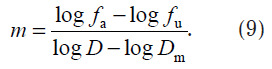



3. The additive model in the analysis of combined drug action. The concept of studying the synergis tic or antagonistic interactions between two drugs is based on the idea of comparing the effect of their combined action f (x)_1,2_ and the sum of the effects of these drugs acting separately f (x)_1_ and f (x)_2_ ; i.e., on an additive model of the following form:

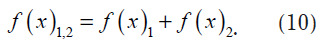



Equation (10) can be represented for the additive model as the following relationship:

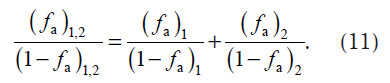



Considering that the chemical reaction has the cor responding order m (Eqs. (5) and (6)), then

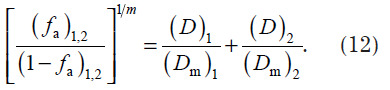



The deviation from additivity under the combined action of chemical agents can be estimated both in terms of their concentration (dose) and the effect they engender, which is reflected in the recommendations of the so-called “Saariselkä Agreement” [21]. In the f irst case, the change in the severity of the effect (ad ditivity of effects, Bliss independence) relative to the additive model is estimated [22] and, in the second case, the change in dose (additivity of doses, Loewe additivity) is estimated [23]. The latter option is more practical in pharmacology and toxicology, as it allows one, in the event of synergism, to calculate the coef f icient of reduction in the concentration of active sub stances while maintaining the initial effect. It is espe cially relevant for pharmaceuticals with pronounced side effects.

A quantitative measure of drugs interactions is the Combination Index (CI) [14]:

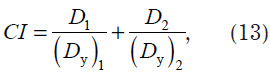



where D_1_ and D_2_ are the concentrations of the drugs 1 and 2 that are used in combination at which the de gree of effect y = f_a_ is achieved; (D_y_ )_1_ and (D_y_ )_2_ are the concentrations of each of the drugs 1 and 2, which individually lead to the same degree of effect y = f_a_ .

The CI value equal to 1 indicates additivity; CI < 1 indicates synergism; and CI > 1 indicates antagonism, respectively.

The (D_y_ )_1_ , (D_y_ )_2_ values for the corresponding y = f_a_ can be obtained either directly from the experiment or theoretically by approximation using Eq. (6) based on sets of experimental dose–response data.

DRI_1_ = (D_y_ )_1_ / D_1_ is the Dose Reduction Index: the index of reduction in the dose of drug 1 in the pres ence of drug 2, an important measure of the influence of drug 2 on the action of drug 1.

In these terms [14]:

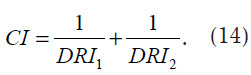



We applied these basic principles to study the ef fect of a targeted anticancer drug on the growth of HER2-positive cells of tumor origin, as well as to eval uate the influence of growth factors and human blood serum on drug efficacy. We investigated this effect on drug action for 17 serum samples from healthy do nors and 19 serum samples from patients with breast cancer and calculated the CI for each sample. These measurements indicate a synergistic interaction be tween the drug and blood serum.

## EXPERIMENTAL

BT474 cells were cultured at 37°C and 5% CO_2_ in RPMI-1640 medium (PanEco, Russia) supple mented with 10–15% FBS (Biosera, France), 2 mM L-glutamine, 4.5 g/L glucose, 1% penicillin–strepto mycin, and 10 ng/mL insulin. SK-BR-3 cells were cultured at 37°C and 5% CO_2_ in RPMI-1640 medi um (PanEco) supplemented with 10% FBS (Biosera), 2 mM L-glutamine, 4.5 g/L glucose, and 1% penicil lin–streptomycin. The following reagents were used in the experiments: trastuzumab (Roche, Switzerland), lapatinib (Sigma-Aldrich, USA), and EGF (SciStore, Russia).

In the SK-BR-3 cells counting experiment, cells were seeded at a concentration of 11,000 cells/mL, 500 µL per well, in 24-well plates (1.86 cm²), resulting in a cell density of 3,000 cells/cm2 [24].

In the BT474 clonogenicity assay, cells were seed ed at 800 cells/mL, 2 mL per well, in 6-well plates (9.026 cm^2^), with a cell density of 174 cells/cm2 [25].

Drugs were added to the cells 16–24 h after seed ing.

Plates with equal numbers of seeded cells were in cubated for 24 h before treatment with EGF, HER2 targeted drugs, or human blood serum samples. After 21 days of incubation, BT474 cells formed colonies. The medium was removed; the cells were fixed with 4% formaldehyde for 10 min, stained with 0.5% crystal violet in 60% methanol and 0.2 × PBS for 15 min, and rinsed with water. Colonies containing more than 50 cells were detected and counted using the openCFU software [26]. Cell survival (CS) was calculated as the ratio between the number of colonies in a drug-con taining well and the number of colonies in the control well without the drug. All the experiments were per formed in at least three independent replicates.

## RESULTS AND DISCUSSION

In clinical practice, antitumor drugs are typically not used as monotherapy but are combined as multi-drug regimens. Therefore, an important exercise is to de termine whether a given drug combination is more effective than using the drugs individually. The pro cess can be tested in vitro using cancer cell lines.

**Part 1. Measuring the interaction between two drugs in the inhibition of cell proliferation**

The Algorithm. To evaluate the interaction between two drugs, the individual drug action parameters (namely, IC_50_ and m for each drug) need to be meas ured, followed by measuring the effect of the drug combination on the cell growth rate and, finally, calcu lation of the Combination Index (CI).

Thus, the algorithm for calculating CI is as follows:

1.1 Estimation of IC_50_ and m for each drug:

1) Incubate cells in the presence of the drug at two or more concentrations and without the drug for sev eral days; count the number of cells (colonies); calcu late the cell survival:

CS = (number of cells after growth with the drug − bkg) / (number of cells after growth without the drug − bkg),

where bkg is the number of cells at the time of add ing the drug.

This measures the cell survival (CS) at drug con centration D.

2) Calculate log(1/CS−1) and the logarithm of the drug concentration logD.

3) Substitute these values into linearized equation (8), from which m and IC_50_ can be calculated:

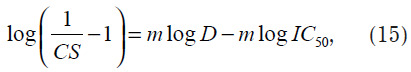



where IC_50_ is the median drug concentration at which cell survival (or clonogenicity) is 50%, and m is a coef ficient indicating the characteristic dependence of cell growth on drug concentration.

Calculate m using the formula:

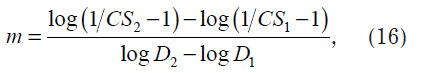



where CS1 and CS2 are cell survival at drug concen trations D1 and D2 , respectively.

Calculate IC_50_ using the formula:

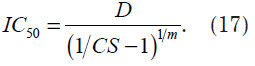



Note: The formula yields the same result with ei ther pair D_1_ , CS_1_ or D_2_ , CS_2_ .

For calculations involving multiple drug concentra tions, we developed an Excel file (see Supplementary materials “Hill Drug Analyzer” sheet “Chou–Talalay”). If the number of measurements is small (2–4), the m and Dm values can also be computed using linear regression (the SLOPE function in Excel, sheet “IC”).

Knowing the IC_50_ and m parameters, the “dose–ef fect” curve can be calculated:

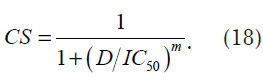



1.2 Evaluation of the degree of interaction between two drugs.

The quantitative assessment of drugs interaction, which determines whether their combined effect is additive, synergistic, or antagonistic, is given by the Combination Index (CI).

To calculate CI:

1) Measure cell survival in the presence of the two drugs together. In this experiment, the control is the action of the drugs separately. Let drug A con centration = D_A_real_ , drug B concentration = D_B_real_ . Measure cell survival CS, defined as the ratio between the number of cells (colonies) grown with drugs and the number of cells (colonies) grown without drugs.

2) Using formula (18), calculate the theoretical con centrations of drugs A and B, D_A_theor_ and D_B_theor_ , corresponding to the observed survival CS: i.e., the survival achieved by the combination treatment:

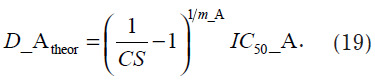

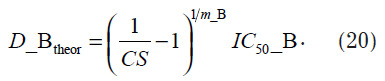





IC_50_ _A and IC_50_ _B are calculated as in section 1.1 from the control drug-alone experiments. It is important that the IC_50_ values come from this particular exper iment, while the parameters m_A and m_B can be used from preliminary experiments.

Calculate the degree of interaction (CI) by substi tuting the obtained values into the formula

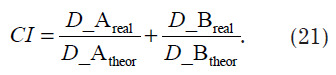



where D_A_real_ and D_B_real_ are the concentrations of drugs A and B in combination.

Since manual calculation is complex, we prepared an Excel spreadsheet where you only need to input D_A_real_ and D_B_real_ and the measured CS values.

Note that if one drug (A) has a weak effect on cell survival (D_Atheor >> IC_50__ A), then

CI = IC_50_ (of drug B alone) / IC_50_ (of drug B with drug A).

This explains the physical meaning of CI: how manyfold more drug B is required if drug A inter feres with its action.

Interpretation:

If CI = 1, the interaction is additive: the drugs act independently.

If CI > 1, the interaction is antagonistic: the drugs interfere with each other.

If CI < 1, the interaction is synergistic: the drugs enhance each other’s action.

**Part 2. Lapatinib and EGF interaction in the inhibition of SK-BR-3 cells proliferation**

The HER receptor family consists of four members: EGFR (epidermal growth factor receptor), HER2, HER3, and HER4 [27]. It is well established that small (7–8 kDa) proteins, growth factors, can bind to these receptors and thus activate them, triggering cell pro liferation [28]. The most abundant growth factor is the epidermal growth factor (EGF).

Breast cancer (BC) is characterized by HER2 over expression in 15–20% of cases [29]; monoclonal hu manized antibody trastuzumab has been approved for its treatment [30, 31]. Lapatinib, a targeted drug that blocks the activation of both HER2 and EGFR, is also widely used in breast cancer therapy.

We measured the effect of HER-targeted drugs, trastuzumab and lapatinib, on the growth rate of HER2-overexpressing (HER2+) cell lines. The BT474 line (derived from ductal carcinoma) and the SK-BR-3 line (derived from squamous cell carcinoma) were used. We further assessed the interactions of these drugs with EGF and human blood serum.

We investigated the influence of EGF or human blood serum on targeted drugs, because they were previously shown to modulate the effects of targeted drugs on A431 cells [32]. For the HER2+ SK-BR-3 and BT474 cells, it was noted earlier that trastuzumab inhibits proliferation both in the absence and presence of EGF, although no quantitation of drug interaction parameters was performed [33].

First, we studied the efficacy of lapatinib as a monotherapy. Before adding the drug, the number of cells in a control well was counted to determine the background value. The drug was added to a standard medium, and after six days of incubation, cell counts were conducted at several drug concentrations. The incubation time was chosen empirically. It was found that when SK-BR-3 cells are incubated for more than six days, the control well reaches confluence and the cell number no longer increases; thus, it can no lon ger be used as a reliable control. When incubated for less than six days, the ratio between the cell number and the initial cell number (on the day the drug was added) decreases. This decrease occurs, because many drugs cause growth arrest without immediate cell de tachment or death; therefore, the ratio between the measured cell number and the cell number before drug addition increases over time.

After counting the cells, we defined the following:

CS = (number of cells after grown in the presence of drug(s), serum, etc. − bkg) / (number of cells after growth without drug − bkg),

where bkg is the number of cells at the time of ad dition of the drug. If CS < 0, it should be considered that CS = 0, indicating that the drug causes cells de tachment from the substrate.

**Fig. 1:**
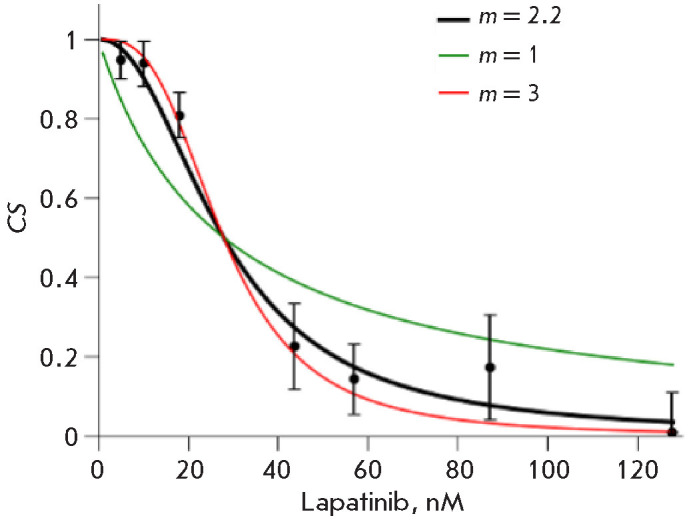
Cell survival (*CS*) of SK-BR-3 cells at growing lapatinib concentrations (0–150 nM). The dependence of cell growth on drug concentration was calculated from three independent replicates, normalized to conditions without drugs. The graph shows the average values (± standard deviations). Three curves represent the dependence of* CS *on drug concentration calculated using formula (18) for *m *= 1, *m *= 3, and *m *= 2.2. The value *m *= 2.2 was calculated using formula (16) and more accurately fits the dependence of cell survival on drug concentration. The calculated *IC*50 = 28 nM (formula (17))

*Figure 1* shows the dependence of SK-BR-3 cell survival on the lapatinib concentration, as well as the dose–effect curves calculated using Eq. (18). One can see that the parameter m is indicative of the steep ness of the decline in the dose–effect curve with a growing lapatinib concentration. When m = 1, the dose–effect curve describes a first-order system; when m < 1, the curve declines more gradually; and when m > 1, cell survival depends more abruptly on the drug concentration.

The point where D = 0, CS = 1, is not included in the plot of CS(D). The measurement at D = 0 is required for normalization (to calculate CS based on the measured cell numbers). Therefore, this point (log(0), 1) should be removed from the logarithmic scale graph to avoid a discontinuity.

**Fig. 2:**
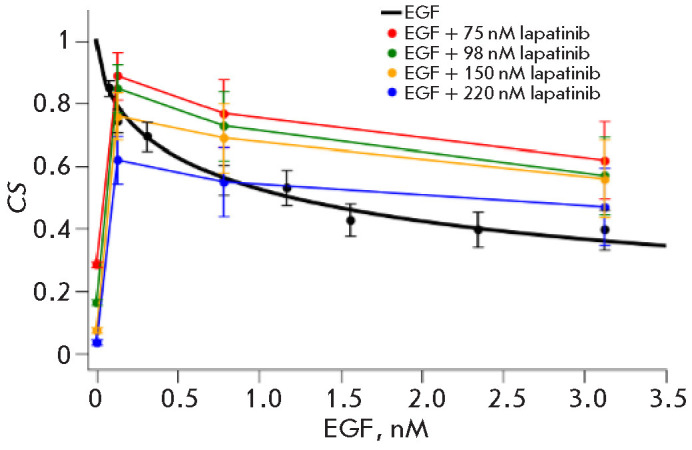
Cell survival (*CS*) of SK-BR-3 cells with growing EGF concentrations without lapatinib (thick black line) and in the presence of the indicated concentrations of lapatinib (75, 98, 150, and 220 nM, thin lines). The dependence of cell growth on drug concentration was calculated from three independent replicates, normalized to conditions without drugs. The graph shows the average values (± standard deviations). For EGF, the calculated parameters are *IC*50 = 1.2 nM, *m *= 0.6. The parameters were calculated using script 1 from the supplementary materials according to formulas (16) and (17). For the EGF–lapatinib interaction, *CI *was calculated using script 2 from the* Supplementary materials*, according to formula (21). The indicated value *CI *= 26.6 is indicative of a strong antagonism between the two compounds

Next, we conducted experiments where SK-BR-3 cells were treated simultaneously with EGF and lapa tinib and the parameters of the drug interaction were calculated (*Fig. 2*). In the absence of EGF, lapatinib ef fectively inhibits cell growth, which is demonstrated on the graph at the point where the EGF concentra tion is zero.

**Part 3. The influence of Cell Growth Rate Measurement Errors on the Estimation of the Inhibition Parameters IC_50_ and m**

of the Inhibition Parameters IC_50_ and m When counting cells or colonies, the measurement er ror is at least 1/√N, where N is the number of cells counted. For instance, to achieve a 10% error, at least 100 cells should be counted. Note that the IC_50_ error cannot be less than the experimental error.

From Eq. (6), we derived the formulas for IC_50_ and m errors:

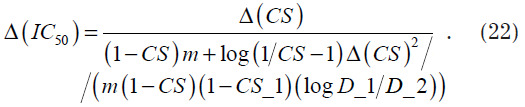



**Fig. 3:**
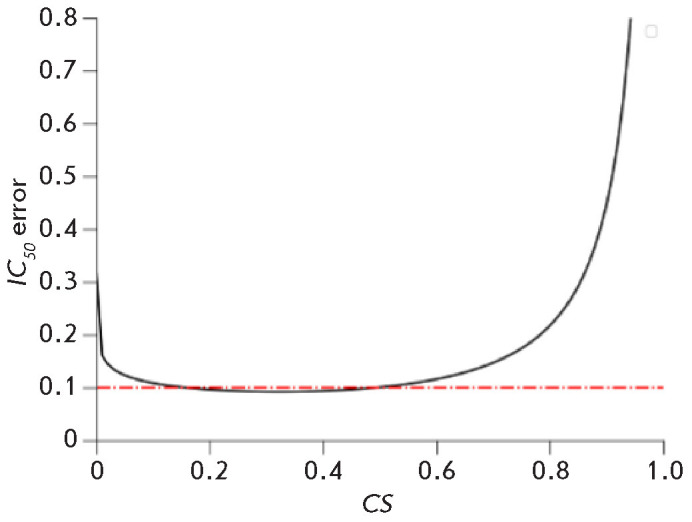
Dependence of the error in calculating the drug* IC*50 on the measured ratio of the number of cells (colonies) in the presence and absence of the drug (*CS*).* CS *error is 0.1

*Figure 3* shows the dependence of IC_50_ determina tion accuracy on CS. For m = 2, the accuracy of IC_50_ determination equals the measurement error of CS at CS = 0.5.

When CS is between 0.25 and 0.75, the accura cy of IC_50_ determination is 10–20% given a CS mea surement error of 10%. At low drug concentrations, when CS is greater than 0.75, the error in measuring CS already exceeds the measurement error twofold. Therefore, measurements at such low drug concentrations are not useful for determining IC_50_ .

At high drug concentrations, when CS is less than 0.25, the measurement error of CS can become large because it is greater than > 1/√N; hence, the number of cells in the control must be sufficiently large. If CS is below 0.25, then counting 100 cells at this CS level (for a 10% error) corresponds to having 400 cells in the control.

The accuracy in determining m is usually about half of the measurement error of CS.

**Part 4. The influence of human blood serum on the effect of trastuzumab on the clonogenicity of BT474 cells**

**Fig. 4:**
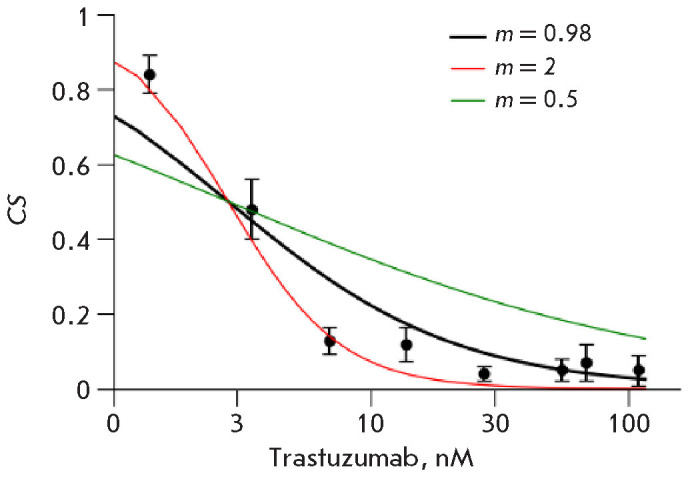
Cell survival (CS) of BT474 cells at growing tras-tuzumab concentrations (0–110 nM). The dependence of the number of colonies on the drug concentration was calculated from three independent replicates, normalized to conditions without the drug. The graph shows the average values (± standard deviations). Three curves represent the calculated dependence of cell survival (*CS*) on drug concentration using formula (18) for *m *= 0.5,* m *= 2, and *m *= 0.98. The value *m *= 0.98 was calculated using formula (16) and more accurately fits the dependence of cell growth on drug concentration. The calculated* IC_50_* = 3.5 nM (formula (17))

We investigated the influence of human blood serum on the effect of the HER2-targeted monoclonal anti body trastuzumab on the clonogenicity of BT474 cells. First, the parameters of trastuzumab’s effect on clo nogenicity were measured in a standard growth me dium (*Fig. 4*), and the IC_50_ and m parameters were calculated. In this semi-logarithmic scale graph, the point (log(0), 1) was removed.

**Fig. 5:**
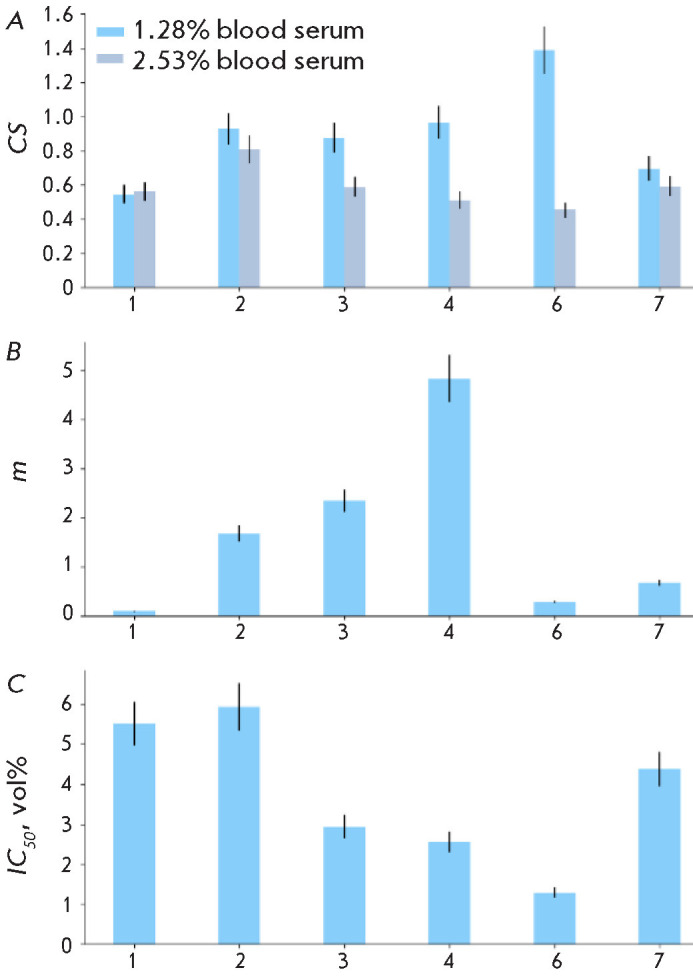
The influence of human blood serum samples on the growth of BT474 cells. (*A*) Clonogenicity of cells in a medium containing human blood serum samples (labeled 1–7) at concentrations of 1.28 and 2.53 vol. %. The bars represent the average clonogenicity for each sample, normalized to control conditions (with 1.28% and 2.53% FBS added). (*B*) The parameter *m*, calculated using script 1 from the *Supplementary materials *according to Eq. (16) for each serum sample. (*C*) The parameter *IC_50_*, calculated using script 1 from the *Supplementary materials *according to Eq. (17). The errors in parameter calculations are due to deviations of the experimental data from the mean values and are shown as error bars

Next, the parameters of the influence of human blood serum samples on clonogenicity without trastu zumab were measured (*Fig. 5*); the IC_50_ and m parameters were also determined for each serum sample.

**Fig. 6:**
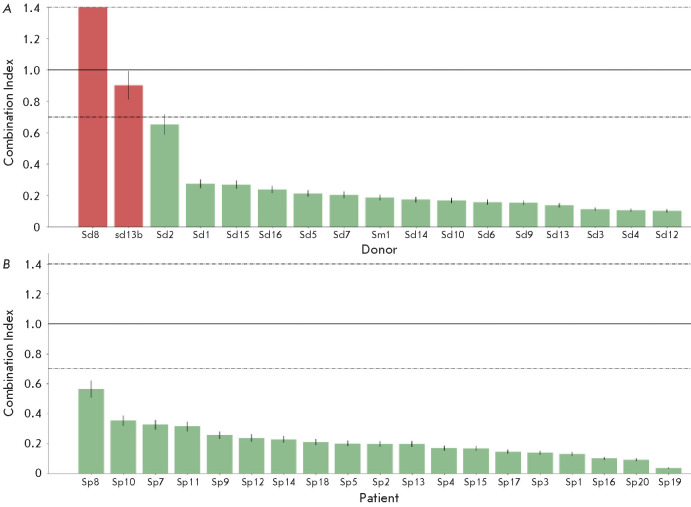
The influence of human blood serum samples on the effect of trastuzumab (3.83 nM) on BT474 cells clonogenicity. The *CI* value was calculated using formula (21) (script 2 from* Supplementary materials*). The bars represent the *CI* values for each blood serum sample of healthy donors (*A*) and breast cancer patients (*B*). The errors in parameters calculations are due to deviations of experimental data from the mean values and are shown as error bars

Knowing the IC_50_ and m parameters for human blood serum samples and trastuzumab, we can deter mine whether the effect of human blood serum on the inhibition of cell growth by trastuzumab is additive, synergistic, or antagonistic. Previously, we measured the influence of human blood serum on the action of trastuzumab on BT474 cells from healthy donors and breast cancer (BC) patients [34]. Based on these data, we calculated the combination index (CI) value for each sample using script 2 from the Supplementary materials according to formula (21) (*Fig. 6*).

We found that out of 17 samples from healthy do nors, 15 demonstrated a synergistic effect on the ac tion of trastuzumab on BT474 cells (CI < 0.65); while all 19 samples from BC patients showed a syner gistic effect on trastuzumab action on BT474 cells (CI < 0.55).

## CONCLUSIONS

Here, we have described an algorithm for a quantita tive measurement of the mutual influence of drugs, specifically calculation of the combination index (CI), and also proposed tools for the calculation of the in dex.

Analysis of experimental data using this algorithm showed that lapatinib inhibits the growth of SK-BR-3 cells with IC_50_ = 28 nM, m = 2.2, and EGF inhibits the growth of SK-BR-3 cells with IC_50_ = 1.2 nM, m = 0.6. The interaction between lapatinib and EGF is antago nistic (CI = 26.6), indicating that the presence of EGF hinders the effect of lapatinib on cancer cell growth.

Trastuzumab inhibits the growth of BT474 cells with IC_50_ = 3.5 nM, m = 0.98, and samples of human blood serum inhibit the growth of BT474 cells with IC_50_ ranging from 1 to 6 vol. %, m ranging from 0.1 to 5 depending on the sample. Human blood serum en hances the inhibitory effect of trastuzumab on the clonogenicity of BT474 cells compared to calf blood serum.

For 17 donor serum samples, the average CI = 0.40, and for 19 serum samples from breast cancer pa tients, the average CI = 0.21. That is, the CI values differ by a factor of 1.9 between the groups. We ob served significant variation in the effects of human blood serum samples both among patients and among healthy donors.

## Abbreviations

BC: breast cancer
EGF: epidermal growth factor
CI: Combination Index
CS: cell sur vival
